# Human PD-L1 overexpression decreases xenogeneic human T-cell immune responses towards porcine kidneys

**DOI:** 10.3389/fimmu.2024.1279050

**Published:** 2024-01-30

**Authors:** Katharina Schmalkuche, Tamina Rother, Sevval Besli, Reinhard Schwinzer, Rainer Blasczyk, Björn Petersen, Constanca Figueiredo

**Affiliations:** ^1^ Institute of Transfusion Medicine and Transplant Engineering, Hannover Medical School, Hannover, Germany; ^2^ Transregional Collaborative Research Centre 127, Hannover Medical School, Hannover, Germany; ^3^ Transplantation Laboratory, Clinic for General, Visceral and Transplantation-Surgery, Hannover Medical School, Hannover, Germany; ^4^ Department of Biotechnology, Institute of Farm Animal Genetics, Friedrich-Loeffler-Institute, Federal Research Institute for Animal Health, Neustadt am Rübenberge, Germany

**Keywords:** xenotransplantation, kidney transplantation, ex vivo organ perfusion, T-cell immune response, genetic engineering, programmed cell death-1 ligand 1 (PD-L1)

## Abstract

Xenotransplantation offers a promising alternative to circumvent the lack of donated human organs available for transplantation. Different attempts to improve the survival of xenografts led to the generation of transgenic pigs expressing various combinations of human protective genes or knocked out for specific antigens. Currently, testing the efficiency of porcine organs carrying different genetic modifications in preventing xenogeneic immune responses completely relies on *in vitro* assays, humanized mouse models, or non-human primate transplantation models. However, these tests are often associated with major concerns due to reproducibility and generation of insufficient data as well as they raise ethical, logistical, and economic issues. In this study, we investigated the feasibility of specifically assessing the strength of human T-cell responses towards the kidneys of wild-type (WT) or transgenic pigs overexpressing human programmed death-1 ligand 1 (hPD-L1) during *ex vivo* kidney perfusion (EVKP). Human T cells were shown to adhere to the endothelium and transmigrate into WT and hPD-L1 kidneys. However, transcript levels of TNF-a and IFN-y as well as cytotoxic molecules such as granzyme B and perforin secreted by human T cells were significantly decreased in the tissue of hPD-L1 kidneys in comparison to WT kidneys. These results were confirmed via *in vitro* assays using renal endothelial cells (ECs) isolated from WT and hPD-L1 transgenic pigs. Both CD4^+^ and CD8^+^ T cells showed significantly lower proliferation rates after exposure to hPD-L1 porcine renal ECs in comparison to WT ECs. In addition, the secretion of pro-inflammatory cytokines was significantly reduced in cultures using hPD-L1 ECs in comparison to WT ECs. Remarkably, hPD-L1 EC survival was significantly increased in cytotoxic assays. This study demonstrates the feasibility of evaluating the human response of specific immune subsets such as human T cells towards the whole xenograft during EVKP. This may represent a robust strategy to assess the potency of different genetic modifications to prevent xenogeneic immune responses and thereby predict the risk of immune rejection of new genetically engineered xenografts.

## 1 Introduction

Kidney transplantation represents the only curative treatment for patients with end-stage kidney failure (1). However, the availability of human kidneys suitable for transplantation is often associated with long periods on the transplant waiting lists. The discrepancy between the number of organs available and the increasing requirement for an organ presents a major obstacle and limits the number of successful kidney transplantations. On average, 18 patients from the waiting list die per day in Europe without receiving the chance of a life-prolonging organ (2).

Xenotransplantation provides a promising alternative to allogeneic transplantation by circumventing the bottleneck regarding available organs. However, despite similar organ size and physiology, genetic differences between species lead to immunological barriers and are a limiting factor for clinical success (1). The establishment of a variety of genetic modifications for xenotransplantation in combination with immunosuppressive and anti-inflammatory agents represents a promising approach to minimizing the risk of rejection (3). Transgenic pigs expressing human protective genes and knocked out for specific immune antigens significantly improved graft survival. In 2001, the first alpha-1,3-galactosyltransferase (GGTA1) deficient pigs were generated, providing a breakthrough success in xenotransplantation of porcine organs by reducing hyperacute rejection (HAR) (4–6). The generation of the first triple KO (GGTA1/Cytidine monophosphate-N-acetylneuraminic acid hydroxylase (CMAH)/β-1,4-acetyl-galactosaminyltransferase 2 (β4GalNT2)) pigs presented an additional milestone in overcoming HAR and acute humoral xenograft rejection (AHXR) (7). However, acute cellular rejection (ACR) remains the major obstacle to a successful xenotransplantation outcome and constitutes a considerable hurdle for long-term graft survival (8). In addition to the involvement of NK cells, macrophages, neutrophils, and B cells, T cells play a leading role in ACR (9, 10). T-cell receptor interaction with MHC, costimulation, and cytokine secretion regulate the activation of naïve T cells, which initiates a programmed differentiation pathway and determines the strength and functionality of the immune responses (11).

Organs of genetically engineered pigs might have the capacity to modulate human cellular immune responses and therefore represent a promising approach to support long-term xenograft survival. Attenuation of T-cell immune responses by preventing T-cell activation could demonstrate a benefit in reducing ACR (10). In cancer research, previous studies have shown that the binding of human programmed death-1 ligand 1 (hPD-L1) to the programmed death-1 (PD-1) receptor on T cells leads to the reduction of PD-1^+^ cell proliferation, inhibition of cytokine secretion, and induction of apoptosis (12). In a xenogeneic setting, Buermann et al. indicated that hPD-L1 peripheral blood mononuclear cells (PBMCs) severe the potential to reduce CD4^+^ T-cell proliferation and induce a low immunogenic, immune-protected status (13). However, the full effect of hPD-L1 overexpression on the porcine renal tissue in preventing human immune responses remains to be evaluated.

So far, potential human immune responses to xenografts carrying different combinations of genetic modifications can only be characterized *in vitro* using cultured human immune cells, in humanized mice models, or non-human primate (NHP) models after xenotransplantation (10, 14). However, the data generated using *in vitro* assays is often insufficient and reflects the immune response against a single target cell type and not against the complete tissue as in the case of transplantation. Studies based on humanized mouse models are strongly dependent on the degree of humanization and capacity to mount reliable immune responses. On the other hand, xenogenic immune responses can be successfully evaluated by transplanting NHPs in a preclinical state, but this strategy is associated with several ethical, logistic, and economic concerns (15, 16). Hence, precise assays enabling the assessment of specific human immune responses towards the xenograft in its complete multi-cell type and structure complexity are highly desirable. *Ex vivo* kidney perfusion (EVKP) has emerged as a promising technology for assessing the quality of kidneys during preservation and has also been shown to serve as a platform for organ conditioning, allowing targeted treatment and quality improvement (17). In this study, we evaluate the feasibilityof specifically assessing T-cell immune responses during EVKP, without the influence of other immune cells. This strategy may allow the characterization of the direct impact of specific genetic modifications in the T-cell immune response using a complete organ as a target and not only specific cell subtypes as in conventional *in vitro* assays. This strategy may enable an initial evaluation of the efficacy of specific genetic modifications and might contribute to reduce and refine the number of animals used for the unavoidable preclinical tests on NHPs.

## 2 Materials and methods

### 2.1 Experimental groups and kidney retrieval

In this study, kidneys from 10 wildtype (WT) (non-perfused WT kidneys (*n* = 3), perfusions of WT kidneys without human T cells (*n* = 3), perfusion of WT kidneys with human T cells (*n* = 4)) and two genetically modified landrace pigs with GGTA1-KO and hPD-L1 overexpression were used. One kidney from each animal was used for perfusion. For organ retrieval, pigs were anesthetized with Propofol (i.v.) and euthanized with pentobarbital (i.v.). After circulatory death, an anterior midline incision was performed and rectus abdominis muscles were separated. The retroperitoneum and peri-renal space are exposed via blunt dissection. Following the dissection of the aorta and inferior vena cava, the kidneys were removed en bloc with these vessels. Kidneys were flushed with 200 mL cold (4°C) Custodiol (Dr. Franz Köhler Chemie GmbH, Bensheim, Germany) and stored on ice during transport to the laboratory.

### 2.2 Isolation of human T cells

PBMCs were isolated from human blood from different healthy donors immediately before the start of perfusion. Briefly, human blood was diluted 1:2 with Dulbecco’s Phosphate Buffered Saline (Lonza, Basel, Switzerland) and centrifuged by density gradient centrifugation in Lymphosep (C. C. Pro, Oberdorba, Germany). Afterward, the CD3^+^ cell population was isolated by negative magnetic bead isolation using the human Pan T Cell Isolation Kit (Miltenyi Biotec Inc., Auburn, California, USA) according to the manufacturer’s instructions. In this study, 3.5x10^7^ T cells were used for perfusion.

### 2.3 Normothermic EVKP

Kidneys were connected to the Kidney Assist^®^ perfusion device (XVIVO B.V., Groningen, Netherlands) via an artery cannula. After kidneys had been warmed up to 37°C for 30 minutes, organs were perfused for 4 hours with 1 L of Williams´s Media E (WME) (Thermo Fisher Scientific, Waltham, Massachusetts, USA) supplemented with 7,15 g HEPES (Sigma Aldrich, Darmstadt, Germany), 50 g/L Bovine Serum Albumin (Sigma Aldrich) and 0,80 g Creatinine (Sigma Aldrich) as previously described (18, 19). T cells were injected into the perfusion system after the perfusate temperature reached 37°C. Perfusion flow, vascular resistance, oxygen saturation, and perfusate temperature were monitored every 30 minutes. After 270 minutes, EVKP was finished and kidneys were flushed with 1 L Custodiol. Non-perfused WT kidneys (*n* = 3), perfusions of WT kidneys without human T cells (*n* = 3), and perfusions only with T cells (*n* = 3) served as controls to WT (n = 4) and hPD-L1 kidneys (n = 2) perfused with T cells.

### 2.4 Histological evaluation

Immediately after perfusion, renal tissues were fixed in 4% paraformaldehyde and embedded in paraffin after 3 days. Tissue slices were stained with hematoxylin and eosin for analyses of renal structure. For immunohistochemistry, tissue slices were stained with anti-human CD3 (UCHT1; BioLegend, San Diego, USA) or CD274 Polyclonal Antibody (Bioss Antibodies, Woburn, Massachusetts, USA) by using the Zytochem Plus HRP Polymer System (Zytomed Systems, Berlin, Germany). Counterstaining was performed using Papanicolaou’s solution and samples were fixed with DPX Mountant (Sigma-Aldrich, St. Louis, Missouri, USA). Afterward, visualization was performed using a Keyence microscope (Keyence, Itasca, Illinois, USA) and samples were quantified via QuPath v0.3.0 bioimage analysis software (open source; https://qupath.github.io/).

### 2.5 Perfusate analyses

#### 2.5.1 Lactate dehydrogenase activity

Perfusate samples were collected at different time points (0, 30, 90, 150, 210, and 270 minutes) of perfusion. Lactate dehydrogenase (LDH) activity in perfusate samples was calculated using the colorimetric Cytotoxicity Detection Kit (LDH) (Roche, Basel, Switzerland) according to the manufacturer’s instructions. The optical density (OD) of the colorimetric assay was used to determine the extent of LDH release.

#### 2.5.2 Lactate levels

Perfusate samples were collected at different time points (0, 30, 90, 150, 210, and 270 minutes) after perfusion start to quantify lactate levels in the perfusate using the Lactate-Glo Assay System (Promega, Madison, Wisconsin, USA) according to the manufacturer’s protocol. For analysis, perfusate samples were diluted 1:50 with DPBS (Lonza), and relative luminescence units (RLU) were calculated using Lumat LB 9507 (Berthold Technologies, Zug, Switzerland) luminometer. L-lactate concentrations were measured by extrapolation using a standard curve.

### 2.6 Quantitative real-time polymerase chain reaction

Pooled tissues collected from 3 regions of the renal cortex and medulla (upper, lower, middle region) were fixed in RNAlater™ Stabilization Solution (Merck, Darmstadt, Germany) immediately after perfusion. Total RNA was isolated using RNeasy Mini Kit (Qiagen, Hilden, Germany) and reverse transcribed to cDNA by High-Capacity cDNA Reverse Transcription Kit (Applied Biosystems, Foster City, California, USA). Transcript levels of human tumor necrosis factor-alpha (TNF-α) (Hs00174128_m1; Thermo Fisher Scientific), interferon-gamma (IFN-γ) (Hs00194264_m1; Thermo Fisher Scientific), granzyme B (GZMB) (Hs00188051_m1; Thermo Fisher Scientific), and perforin (Hs00169473_m1; Thermo Fisher Scientific) were measured by utilizing TaqMan Gene Expression Master Mix (Thermo Fisher Scientific). All samples were analyzed in triplicates using the StepOnePlus Real-Time PCR system and results were evaluated by StepOnePlus Software v2.3 (Applied Biosystems). GAPDH (Hs02786624_g1, Thermo Fisher Scientific) was used as an endogenous control for the normalization of mRNA levels.

### 2.7 Isolation of renal endothelial cells

Biopsies were collected from three regions of the kidney (upper, lower, middle region) of unperfused WT or unperfused hPD-L1 renal cortex and medulla. Biopsies were pooled and digested with Collagenase Type I (Sigma-Aldrich) to obtain a single-cell suspension. Cells were cultured in endothelial cell growth medium (EGM-2) (Lonza) on gelatin-coated plates. EC phenotyping was performed by analyzing CD31 and CD144 expression using APC/Cyanine7 anti-human CD31 (WM59; BioLegend) and Alexa Fluor^®^ 647 mouse anti-human CD144 (55-7H1; BD Biosciences, Franklin Lakes, New Jersey, USA) antibodies. PD-L1 expression was evaluated using APC anti-human CD274 antibody (29E.2A3; BioLegend). Data were evaluated by BD FACSCanto™ II Clinical Flow Cytometer System (BD Biosciences) and results were analyzed using FlowJo software v10.6.2 (BD Biosciences).

### 2.8 Human T-cell proliferation assay

24 hours before the start of cell co-culturing (day 0), 2x10^4^ target cells (WT and hPD-L1 ECs) were seeded in triplicates onto a 96-well plate. On day 1, 2x10^5^ T cells from four healthy donors were labeled with the cell proliferation dye efluor 670 (Thermo Fischer Scientific). Afterward, T cells were added in a 10:1 (E: T) ratio to the ECs and cultured in RPMI 1640 Medium (Lonza) supplemented with 5% AB serum (c.c.pro GmbH) and interleukin (IL)-2 (100 and U/mL; Prepotech, New Jersey, USA). After 7 days of co-culturing, the experiment was finished and T cells were stained with FITC anti-human CD3 antibody (UCHT1; BioLegend), APC/Cyanine7 anti-human CD4 antibody (SK3; BioLegend), and PE anti-human CD8 antibody (SK1; BioLegend). T-cell proliferation rates were evaluated by comparing proliferation values of day 0 with day 7 using BD FACSCanto™ II Clinical Flow Cytometer System (BD Biosciences) and results were analyzed using FlowJo software v10.6.2 (BD Biosciences).

### 2.9 Real-time cytotoxicity assay

24 hours before the experiment started, 2x10^4^ target cells (WT and hPD-L1 ECs) were seeded in duplicates onto microtiter plates (E-Plates^©^; Agilent Technologies, California, USA). After achieving a confluent EC monolayer, 2x10^5^ T cells were isolated from three different donors and added in a ratio of 10:1 (E:T) to the target cells. Cells were cultured in RPMI 1640 medium (Lonza) supplemented with 5% human serum AB (c.c.pro GmbH) and IL-2 (100 U/mL) (Prepotech) for 140 hours. Cell proliferation as a function of real-time changes in electrical impedance, also referred to as cell index, was continuously monitored using the xCELLigence Real-Time Cell Analyzer (Agilent Technologies).

### 2.10 Cytokine multiplex analyses

Pro-inflammatory cytokine profile indicating a xenogenetic T-cell response was determined in the real-time cytotoxicity assay´s supernatant using the MILLIPLEX Human Cytokine/Chemokine/Growth Factor Panel A Magnetic Bead Panel (Merck KGaA, Darmstadt, Germany). Briefly, secretion levels of human IFN-γ, IL-8, interferon gamma-induced protein 10 (IP-10), granulocyte-macrophage colony-stimulating factor (GM-CSF), IL-5, IL-10, IL-1b, and IL12p70 were quantified in 25 µL centrifuged perfusate samples collected at the end of the assay (time point 140 hours) and measured using Luminex^®^ 100/200 analyzer (Luminex Corp., Austin, Texas, USA). Standard and sample preparations were performed according to the manufacturer’s instructions. Cytokine levels were calculated using the Xponent software version 3.1 (Luminex Corp.).

### 2.11 Statistical analyses

All data are presented as mean ± standard deviation. For comparison between two groups, the student’s t-test was used. One-way ANOVA with multiple comparisons was applied to compare data with one variable between more than two groups. Two-way ANOVA was used for comparisons of data with two categorical variables between more than two groups. *p* < 0.05 were considered significant and defined as *p < 0.05, ** *p* < 0.01, ***p < 0.001, and ****p < 0.0001. All statistical analyses were performed using GraphPad Prism version 8 software (GraphPad Software Inc, San Diego, California, USA).

## 3 Results

### 3.1 hPD-L1 expression on renal tissue of WT and transgenic pigs

**Figure 1:**
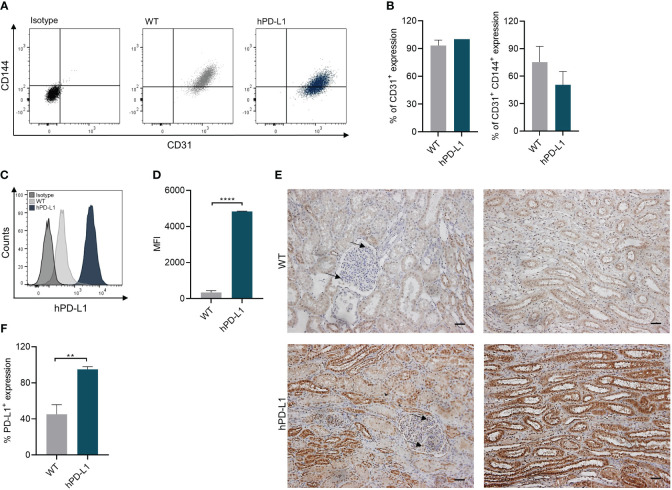
Endothelial cell (EC) isolation from WT and hPD-L1 kidneys. **(A)** Representative dot plots of CD31^+^ and CD144^+^ expression on renal ECs. **(B)** Mean and standard deviation of CD31^+^ and CD31^+^ CD144^+^ expression on ECs isolated from WT (*n* = 3) and hPD-L1 (*n* = 2) kidneys. **(C)** Representative histogram shows PD-L1 expression on WT and hPD-L1 ECs. **(D)** MFI and standard deviation of PD-L1 expression on ECs (WT: *n* = 3; hPD-L1: *n* = 2). **(E)** Immunohistochemistry staining demonstrated representative PD-L1 expression on WT and hPD-L1 kidney tissues (Scale bar: 50µm). Arrows indicate endothelial cells. **(F)** Mean percentage and standard deviation of PD-L1 expression on immunohistochemistry stained tissues (WT: *n* = 4; hPD-L1: *n* = 2). Statistical significance was evaluated using an unpaired t-test (**p < 0.01; ****p < 0.0001).

ECs play crucial roles during graft rejection by several mechanisms including antigen presentation to circulating T cells, or by triggering inflammatory processes and thrombosis (20). Therefore, we confirmed the overexpression of hPD-L1 on renal ECs isolated from the transgenic pigs. Expression of typical markers such as CD31 and CD144 on isolated WT (CD31: 93.30 ± 4.67%; CD31^+^ CD144^+^: 75.37 ± 14.06%) and hPD-L1 ECs (CD31: 99.86 ± 0.19%; CD31^+^ CD144^+^: 46.47 ± 10.71%) showed no significant differences between the groups (**Figures 1A, B**). Remarkably, flow cytometry analyses revealed significantly (*p* < 0.0001) increased PD-L1 expression on hPD-L1 (MFI: 4832.50 ± 11.50) in comparison to WT (MFI: 342.00 ± 80.70) ECs (**Figures 1C, D**). Accordingly, immunohistochemically quantification showed significantly (*p* < 0.01) increased PD-L1 expression in the renal tissue of hPD-L1 (94.94 ± 2.15%) in comparison to WT (45.13 ± 9.17%) pig-derived tissues (**Figures 1E, F**).

### 3.2 Effect of T cells on EVKP parameters

**Figure 2:**
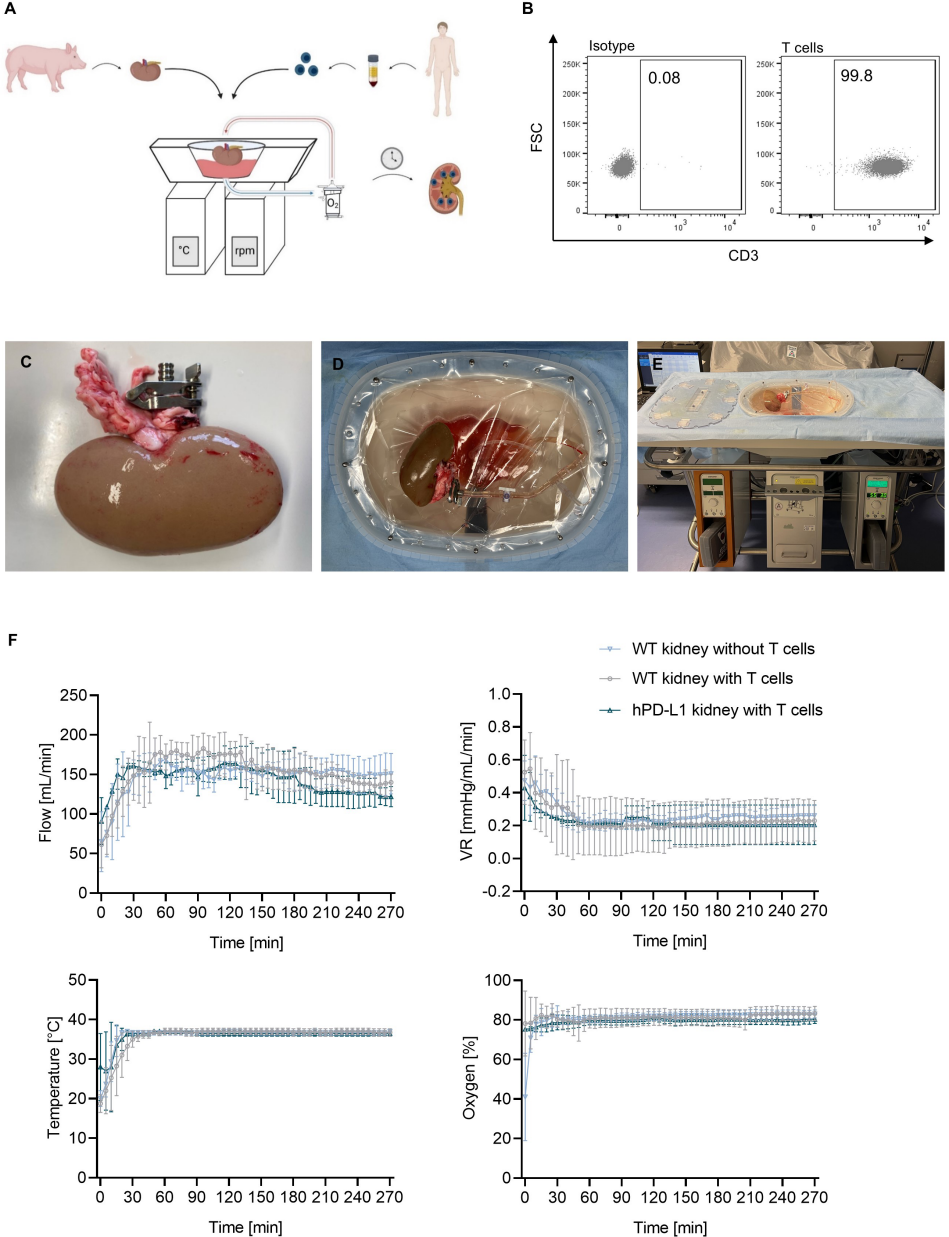
Normothermic *ex vivo* kidney perfusion (EVKP) with human T cells. **(A)** Schematic representation of the EVKP system. The figure illustrates the kidney retrieval from a donor pig, the isolation of the T cells from human blood, and the main components of the perfusion system (perfusion reservoir, thermo unit, pump unit, oxygenator). **(B)** Representative dot plots show CD3^+^ expression on human T cells after isolation. **(C–E)** Representative pictures of the kidney during perfusion. **(C)** Picture displays the clamp placed in the renal artery, **(D)** the arterial connection of the kidney to the perfusion system, and **(E)** the kidney perfusion system. **(F)** Graphs display the monitored flow rate, vascular resistance (VR), temperature, and oxygen partial pressure of wildtype (WT) kidney perfusions without T cells (*n* = 3), WT kidney perfusions with T cells (*n* = 4), and programmed death ligand-1 (hPD-L1) kidney perfusions with T cells (*n* = 2). Graphs show means and standard deviations.

EVKP represents a promising opportunity to provide optimal organ preservation and quality assessment between organ retrieval and transplantation (17). In this study, we specifically evaluated *ex vivo* human T-cell immune responses targeting the renal endothelium during normothermic EVKP as an alternative method to assessment after transplantation. After a 30-minute warm-up period of the perfusion solution to 37°C, the perfusion temperature was kept constant at normothermic temperatures of 36-37°C for the entire perfusion period. WT kidneys perfused without T cells, WT kidneys perfused with T cells, and hPD-L1 kidneys perfused with T cells reached average flow rates of 158.93 ± 27.75 mL/min, 153.34 ± 15.35 mL/min, and 145.45 ± 19.93 mL/min, respectively, with corresponding vascular resistance (VR) values of 0.22 ± 0.15 mmHg/mL/min, 0.25 ± 0.05 mmHg/mL/min, and 0.21 ± 0.07 mmHg/mL/min. During the entire perfusion, oxygen saturation in the perfusate was maintained at constant values of 81.41 ± 4.06%, 81.94 ± 2.10%, and 80.01 ± 1.92%, respectively. Despite initial variations in perfusion parameters during the warm-up phase, no significant differences were observed in flow rate, VR, perfusion temperature, and oxygen saturation during normothermic EVKP (**Figures 2A–F**).

### 3.3 Perfusion with human T cells does not induce tissue damage

**Figure 3:**
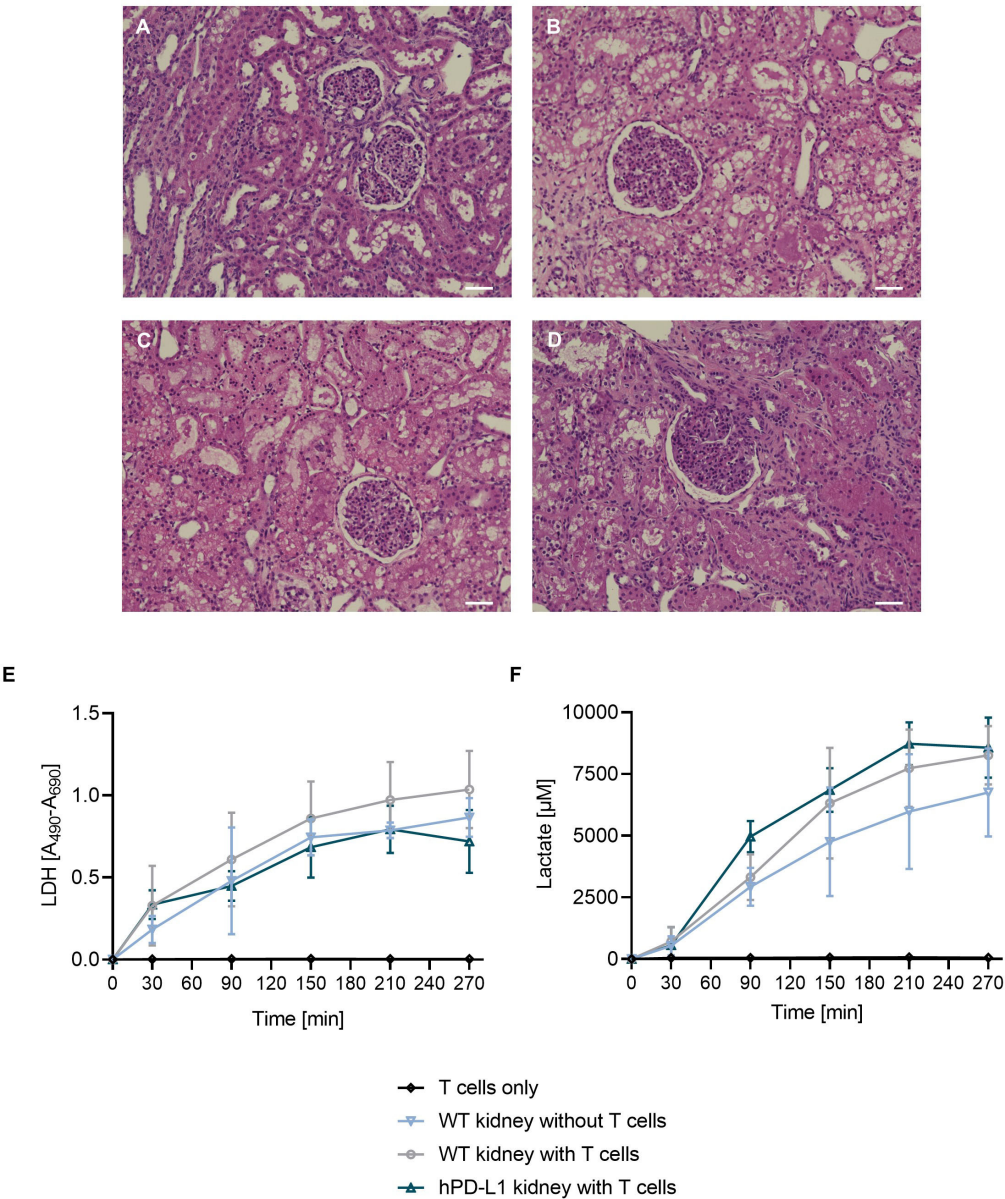
Quality assessment of the kidney after EVKP. **(A–D)** Histological analysis of kidneys after EVKP. Representative pictures of hematoxylin and eosin-stained renal cortex: **(A)** unperfused WT kidney, **(B)** WT kidney perfused without T cells, **(C)** WT kidney perfused with xenoreactive human T cells, and **(D)** hPD-L1 kidney perfused with xenoreactive T cells. Scale bar 50 µm. **(E)** Lactate dehydrogenase (LDH) activity and **(F)** lactate levels were quantified in perfusates at different time points (0, 30, 90, 150, 210, and 270 minutes). Graphs represent means and standard deviations of perfusions only with T cells (*n* = 3), WT kidney perfusions without T cells (*n* = 4), WT kidney perfusions with T cells (*n* = 3), and hPD-L1 kidney perfusions with T cells (*n* = 2).

Histological analyses were performed to evaluate tissue integrity after EVKP. The histopathological findings suggested no significant difference between kidney perfusions with and without T cells, or between T-cell perfusions of WT and hPD-L1 kidneys. However, all perfused kidneys exhibited mild dilatation of Bowman’s capsule and potentially reversible moderate acute intratubular injury with overall intact renal morphology (**Figures 3A–D**).

LDH activity levels are considered a marker to assess tissue integrity (21, 22). During EVKP, no significant differences in LDH levels were observed in perfusion solution of kidney perfusions without T cells (T_270_: 0.87 ± 0.10), WT kidney perfusions with T cells (T_270_: 1.04 ± 0.20), and hPD-L1 kidney perfusions with T cells (T_270_: 0.72 ± 0.14) compared to the LDH activity level absence detected in perfusions only with T cells (**Figure 3E**).

Lactate levels are commonly used as a marker to evaluate tissue integrity and indicate signs of acute injury (23). An increase in the lactate concentration in the perfusion solution was detected during the perfusion of WT kidneys without T cells as well as WT and hPD-L1 kidney perfusions with T cells. As expected, no lactate increase could be detected in control runs only with T cells over time. In contrast, lactate concentrations of kidney perfusions without T cells (T_270_: 6756.84 ± 1461.59 µM), WT kidney perfusions with T cells (T_270_: 8260.99 ± 1025.62 µM), and hPD-L1 kidney perfusions with T cells (T_270_: 8272.83 ± 50.13 µM) increased throughout the perfusion time (**Figure 3F**). The results suggest that xenogeneic T cells do not significantly affect kidney integrity or tissue injury during EVKP.

### 3.4 hPD-L1 ECs induce weaker xenogeneic T-cell immune responses

**Figure 4:**
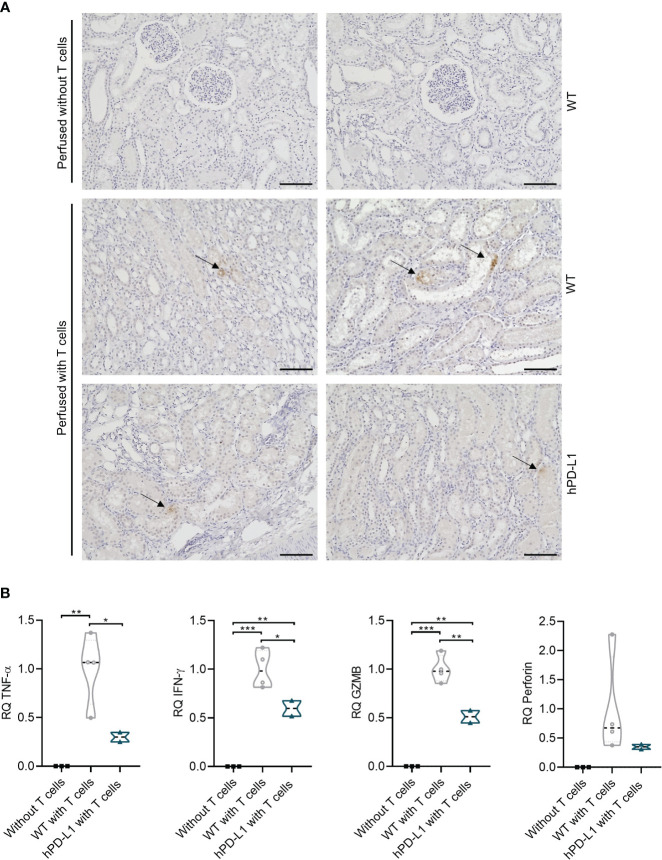
Evaluation of T-cell infiltration in renal tissue and assessment of xenogeneic T-cell immune response after EVKP. **(A)** CD3^+^ immunohistochemistry staining of the perfused kidney without T cells and with human T cells (WT and hPD-L1) after the end of perfusion. Arrows point to the infiltrated T cells (Scale bar: 100µm). **(B)** Relative quantification (RQ) of tumor necrosis factor-alpha (TNF-α), interferon-gamma (IFN-γ), granzyme B (GZMB), and perforin transcript levels detected in kidney tissue perfused without T cells (*n* = 3), WT kidney tissue perfused with T cells (*n* = 4), and hPD-L1 kidney tissue perfused with T cells (*n* = 2). Statistical significance was evaluated using one-way ANOVA (*p < 0.05, **p < 0.01, and ***p < 0.001).

Immunohistochemical analyses of perfused tissue allowed the detection of T-cell transmigration into the tissue. Whereas no T-cell infiltration occurred in the tissue perfused without T cells, CD3^+^ cells were detected in the tissue perfused with T cells from both WT and hPD-L1 kidneys after the end of the perfusion. The transmigrated T cells were predominantly localized in the renal tubule after four hours of perfusion, however, individual T cells had already infiltrated into the tissue (**Figure 4A**). This data shows that during EVKP, T cells are capable to adhere and transmigrate into the renal tissue where they might respond to it.

The transcript levels of human cytokines including TNF-α and IFN-γ, as well as GZMB, and perforin were measured to investigate xenogeneic T-cell responses. Human T cells were demonstrated to respond specifically to porcine kidney tissue, as evidenced by increased T-cell transcript levels of cytokines and cytotoxic molecules in perfused WT and hPD-L1 porcine kidney tissues compared to porcine tissues perfused without T cells, where they were not detectable. TNF-α, IFN-γ, GZMB, and perforin transcript levels of hPD-L1 kidneys were reduced by 70.08 ± 4.94% (*p* < 0.01), 40.35 ± 8.00% (*p* < 0.01), 48.92 ± 6.35% (*p* < 0.001), and 65.03 ± 4.15% (non-significantly), respectively, in comparison to WT kidneys (99.99 ± 31.59%, 99.96 ± 16.78%, 99.96 ± 12.13%, 99.95 ± 74.83%) (**Figure 4B**). These data suggest that hPD-L1 kidneys may induce weaker xenogeneic T-cell immune responses.

### 3.5 hPD-L1 overexpression on porcine ECs shows a protective effect against xenogeneic T-cell responses

**Figure 5:**
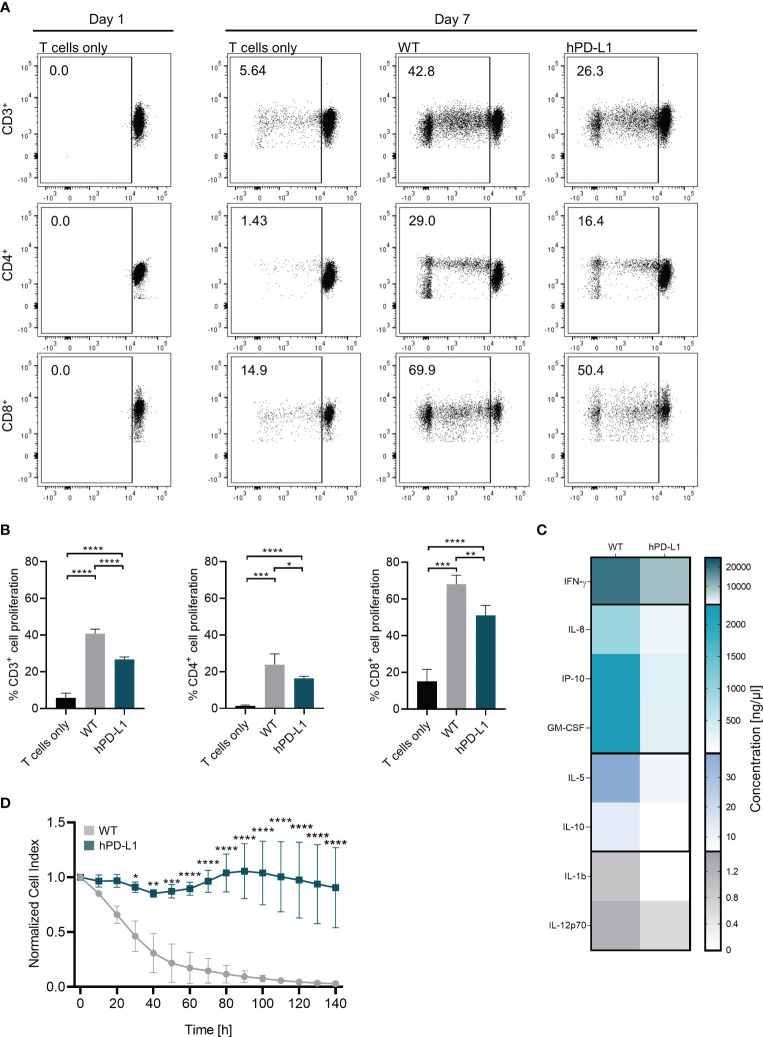
hPD-L1 overexpression on porcine ECs shows a protective effect against xenogeneic T-cell responses. **(A)** Representation of CD3^+^, CD4^+^, and CD8^+^ T-cell proliferation on day 1 and day 7. Proliferation rates of T cells co-cultured without target cells (T cells only) and xenoreactive human T cells isolated from four donors co-cultured with WT and hPD-L1 ECs were evaluated. **(B)** Mean and standard deviation of CD3^+^, CD4^+^, and CD8^+^ T-cell proliferation of T cells alone or exposed to WT and hPD-L1 ECs. Statistical significance was evaluated using one-way ANOVA (*p < 0.05, **p < 0.01, ***p < 0.001, and ****p < 0.0001). **(C)** Heat map represents cytokine release profile after exposure of human T cells to WT and hPD-L1 ECs for 140 hours. Color saturation represents the average values of the concentrations (*n* = 3) of IFN-γ, interleukin 8 (IL-8), interferon gamma-induced protein 10 (IP-10), granulocyte-macrophage colony-stimulating factor (GM-CSF), IL-5, IL-10, IL-1b, and IL-12p70. **(D)** Normalized cell index of WT and hPD-L1 ECs incubated for 6 days with human T cells (*n* = 3). Statistical significance was evaluated using two-way ANOVA (*p < 0.05, **p < 0.01, ***p < 0.001, and ****p < 0.0001).

Antigen-specific immune responses are usually associated with an increase in T-cell proliferation rates (24). We have assessed the capacity of human helper or cytotoxic T-cell subpopulations to proliferate after exposition to EC isolated from WT or hPD-L1 pigs. In this xenogeneic setup, significantly reduced CD4^+^ helper and CD8^+^ cytotoxic T-cell proliferation was observed when hPD-L1 ECs were analyzed (CD4^+^: 16.38 ± 0.96%, *p* < 0.05; CD8^+^: 51.10 ± 4.70%, *p* < 0.01) in comparison to the proliferation rates detected with WT ECs (CD4^+^: 23.80 ± 5.09%; CD8^+^: 68.08 ± 4.21). A similar effect was observed in cultures using both CD4^+^ and CD8^+^ T cells (i.e. CD3^+^ T-cell populations). While only 26.68 ± 1.26 (*p* < 0.0001) of CD3^+^ cells proliferated in the presence of hPD-L1 ECs, 40.78 ± 2.12% CD3^+^ cells proliferated after exposure to WT ECs (**Figures 5A, B**).

Cytokines are important mediators of immune responses after transplantation (25). Levels of cytokines detected in the cell culture supernatants of T cells incubated with hPD-L1 ECs were lower than those measured in WT ECs: IL-1β: 6.45 ± 8.47 ng/µL vs. 0.98 ± 1.38 ng/µL, IL-5: 37.97 ± 17.33 ng/µL vs. 6.91 ± 4.29 ng/µL, IL-8: 2368.43 ± 2456.10 ng/µL vs. 497.71 ± 501.51 ng/µL; IL-10: 28.92 ± 23.55 ng/µL vs. 9.79 ± 7.31 ng/µL; IL-12p70: 2.32 ± 1.60 ng/µL vs. 2.19 ± 2.48 ng/µL; GM-CSF: 1839.99 ± 798.28 ng/µL vs. 360.34 ± 267.17 ng/µL; IFN-γ: 1838.99 ± 798.28 ng/µL vs. 360.34 ± 268.17 ng/µL, and IP-10: 1720.55 ± 955.90 ng/µL vs. 1004.77 ± 1002.40 ng/µL (**Figure 5C**).

In addition, assessment of T-cell-mediated cytotoxicity is critical for evaluating the capacity of the xenogeneic immune response to injure the graft, as even a small number of T cells may lead to organ rejection (26, 27). Compared to WT ECs, hPD-L1 ECs co-cultured with human T cells exhibited higher survival rates (CI, Cell index) over time (140 hours). After 30 hours, the survival rate of hPD-L1 ECs (CI: 0.91 ± 0.04, *p* < 0.05) was significantly increased in comparison to WT ECs (CI: 0.46 ± 0.11). This effect was even more pronounced after 60 hours (hPD-L1 CI_60-140_: 0.98 ± 0.23 vs. WTCI_60-140_: 0.09 ± 0.07, *p* < 0.0001) (**Figure 5D**). Altogether, these data indicate that hPD-L1 overexpression on renal endothelial cells induces significantly weaker immune responses and results in protection against T-cell xeno-cytotoxicity.

## 4 Discussion

Xenotransplantation of porcine organs represents a promising approach to circumvent the shortage of human organs available for transplantation. Breakthrough advances in the field of xenotransplantation have been made by the revolutionized CRISPR-Cas9 technology, which allows the development of multigene-modified pigs such as triple KO (GGTA1/CMAH/β4GalNT2) pigs to overcome HAR and AHXR (28). In 2021, the first kidney xenotransplantations from pig-to-human were performed. Two genetically modified kidneys were transplanted into brain-dead patients observing graft survival of 54 hours (29). In January 2022, the first heart xenotransplantation from pig-to-human was performed. The patient survived two months with the xenogeneic transplant (30).

Currently, the pre-clinical evaluation of the efficiency of novel genetic modifications introduced to pigs in preventing human immune responses relies on the use of NHPs. However, on the one hand, the application of NHPs is associated with relevant ethical and moral evaluation in terms of social, health, religious, legal, and regulatory considerations (15), and on the other hand, NHPs do not represent identically pig-to-human coagulation and immune responses due to the species intrinsic genetic differences. First, macaques carry a “hypercoagulable” phenotype, which can lead to increased coagulopathy compared to humans. Also in contrast to humans, NHP and pigs express the N-glycolylneuraminic acid (Neu5Gc) and therefore NHP does not form specific anti-pig Neu5Gc antibodies as it occurs after xenotransplantation in humans (31). The application of *in vitro* immunological assays to establish and test novel genetic modifications is highly desirable. However, such assays often focus on the use of a single cell type and do not represent the level of organ complexity in cell composition and 3D structure, making them prone to deliver insufficient data.

EVKP emerged as a novel strategy for organ preservation with the potential to reduce storage damage, improve graft assessment, and potentially contribute to graft survival after transplantation (32). EVKP allows the maintenance of the organ under physiological parameters supported by continuous oxygen delivery, and pulsatile flow through the renal vasculature at normothermic conditions (33, 34). These physiological environments were shown to be appropriate for evaluating the human xenogeneic T-cell immune response *ex vivo*. This assay may be used as a first assessment of the T-cell response towards genetically engineered pig kidneys not only to elucidate cellular and molecular mechanisms but also to allow the reduction of animals and refinement of NHP pre-clinical studies. It should be mentioned, that this strategy alone is not sufficient to replace the preclinical studies, as they provide further indispensable results in terms of longer evaluation time, graft function, and safety.

Recently, *ex vivo* organ perfusion (EVOP) has gained plenty of attention as a model to evaluate human xenogeneic immune response. Previous studies using *ex vivo* heart perfusion with human whole blood showed an increase of cytokines such as IL-2, IL-4, and IFN-γ as well as cytotoxic molecule secretion such as GZMB and perforin by T-cell subsets (35). Moreover, pig kidneys have been perfused with human whole blood (36) or human peripheral blood lymphocytes (37). In this study, we used EVKP as a model perfusing porcine kidneys with freshly isolated human T cells to assess the pig-to-human T cell-mediated xenogeneic immune responses. This allows us to evaluate precisely the impact of specific genetic modifications in the pig kidney on human xenogeneic T-cell responses without the interference of other immune cell subpopulations that might have been activated due to the perfusion conditions.

The graft endothelium is the first immune checkpoint between the recipient’s immune system and the renal graft (38). However, recent studies also indicate that renal proximal tubular epithelial cells may also be directly recognized by T cells, which may immediately contribute to rejection (39, 40). Our immunohistochemistry analyses after EVKP indicate the presence of human T-cell focal adhesions in different kidney regions where the recognition of xenoantigens may occur. Our results are consistent with previous studies in rats examining the levels of T-cell infiltration after transplantation of PD-L1-expressing porcine B cells under the renal capsule. Compared to the mock-transfected control cells, which showed scattered to moderate T-cell infiltration after 7 days, the number of infiltrated T cells remained low in the rats transplanted with hPD-L1 cells (41).

To assess the strength of the T-cell immune responses we have evaluated the up-regulation of immunomodulators and cytotoxic molecules. Cytokines such as TNF-α or IFN-γ play a crucial role in the processes such as immune modulation and inflammatory response. TNF-α is both a pro-inflammatory and an anti-inflammatory cytokine secreted by effector CD4^+^ and CD8^+^ T cells (42, 43). Low TNF-α concentrations contribute to cell survival, differentiation, and proliferation, however, excessive activation of TNF-α signaling is often associated with chronic inflammation (44). IFN-γ is secreted by T-helper cells and contributes to the activation of macrophages (45). Perforin and granzymes are important effector molecules of cytotoxic T cell-mediated cell death. The cytotoxic granules of T cells contain the pore-forming protein perforin and serine proteases (granzymes) (46), which can be considered markers for rejection (47). Accordingly, this study demonstrated the feasibility of assessing the activation of T cells by the pig kidney based on the upregulation of TNF-α, IFN-γ, GZMB, and perforin transcript levels during EVKP. Remarkably, we could demonstrate that transcript levels of those molecules were downregulated during hPD-L1 kidney perfusion compared to the WT kidneys, suggesting a potential protective effect of hPD-L1 against acute cellular rejection mediated by xenoreactive T cells.

The value of evaluating human T-cell responses during EVKP relies on the feasibility of assessing T-cell recognition, activation, and functionality in a nearly physiological setup. However, several parameters associated with the perfusion such as pressure or flow rates may influence the T-cell response towards the organ. Furthermore, a limitation in the assessment of the feasibility of evaluating such responses was the number of hPD-L1 transgenic pigs available for this study. Therefore, we performed additional *in vitro* studies to confirm the results obtained during EVKP with human T cells.

Previous studies have indicated that PD-L1 overexpression inhibits the proliferation of human xenogeneic CD4^+^ T cells and induces T-cell apoptosis (8, 13, 48, 49). Accordingly, our results showed reduced CD4^+^ T-cell proliferation rates after exposure to porcine renal ECs. In addition, reduced CD8^+^ T-cell proliferation was also observed.

Previously, we and others have shown that the cytokine secretion profile of T cells is associated with the strength of their response to target cells. In addition, secretion of pro-inflammatory cytokines such as IFN-γ or IL-1β was shown to correlate with kidney rejection. Also, in the pig-to-primate xenotransplantation setting, specific cytokines such as IFN-γ were demonstrated to be relevant systemic inflammatory factors that might contribute to the loss of xenograft function (50–52). On the other hand, decreased levels of cytokines such as IL-6, IL-10, IL-12, TNF-α, and IFN-γ have been associated with increased immunologic tolerance, reduced risk of acute rejection, and thus prolonged graft survival (53, 54). Our results indicate that the overexpression of hPD-L1 in porcine renal ECs significantly decreases the T-cell cytokine secretion. This suggests that the overexpression of hPD-L1 might contribute to xenograft survival.

PD-L1 expression is known to compromise T cell-mediated cytotoxicity against tumors (55). In the field of xenotransplantation, *in vitro* studies using a porcine B cell line overexpressing PD-L1 were shown to induce lower T-cell activation and cytotoxicity (48). Interestingly, in the pig-to-rat cell transplantation model, overexpression of PD-L1 was also demonstrated to induce weaker antibody-mediated immune responses (41). Our results using ECs isolated from the kidneys of hPD-L1 transgenic pigs confirmed that the overexpression of hPD-L1 contributes to the attenuation of T-cell cytotoxicity.

Unfortunately, α-Gal knockout pig kidneys were not available for this study and we only had access to two PD-L1 kidneys. Nevertheless, this study showed the feasibility of investigating T-cell immune responses during EVKP and that by using two types of kidneys (WT vs. PD-L1) alteration in the strength of the immune responses could be detectable. Furthermore, the responses observed during EVKP were in accordance with previously published data and with the results obtained during the *in vitro* assays performed in this study.

In summary, we represented the feasibility of evaluating the strength of human xenogeneic T-cell immune responses towards the pig kidney in its original complexity at cellular and structural levels during EVOP. Remarkably, we demonstrated that the immunogenicity of hPD-L1 kidneys for xenoreactive T cells was reduced compared with WT kidneys in both EVKP and *in vitro* assays. Hence, EVOP may be used in the future as a robust platform, ethically justifiable, and cost-effective approach to investigate additional genetic modifications that might contribute to the success of xenotransplantation.

## Data availability statement

The original contributions presented in the study are included in the article/supplementary materials, further inquiries can be directed to the corresponding author/s.

## Ethics statement

The studies involving humans were approved by Ethic Committee of the Hannover Medical School. The studies were conducted in accordance with the local legislation and institutional requirements. The participants provided their written informed consent to participate in this study. The animal study was approved by Niedersächsische Landesamt für Verbraucherschutz und Lebensmittelsicherheit. The study was conducted in accordance with the local legislation and institutional requirements.

## Author contributions

KS: Conceptualization, Formal analysis, Data curation, Investigation, Methodology, Visualization, Writing – original draft. TR: Writing – review & editing. SB: Investigation, Writing – review & editing. RS: Supervision, Writing – review & editing, Investigation. RB: Resources, Writing – review & editing. BP: Resources, Conceptualization, Writing – review & editing. CF: Conceptualization, Formal analysis, Funding acquisition, Investigation, Methodology, Project administration, Supervision, Writing – original draft, Writing – review & editing.
